# Toll-like receptor 4 mediates microglial activation and production of inflammatory mediators in neonatal rat brain following hypoxia: role of TLR4 in hypoxic microglia

**DOI:** 10.1186/1742-2094-10-23

**Published:** 2013-02-06

**Authors:** Linli Yao, Enci Mary Kan, Jia Lu, Aijun Hao, S Thameem Dheen, Charanjit Kaur, Eng-Ang Ling

**Affiliations:** 1 Key Laboratory of the Ministry of Education for Experimental Teratology, Shandong Provincial Key Laboratory of Mental Disorders, Department of Histology and Embryology, Shandong University School of Medicine 250012, Jinan, China; 2 Defense Medical and Environmental Research Institute, DSO National Laboratories, 27 Medical Drive, Singapore 117510, Singapore; 3 Department of Anatomy, Yong Loo Lin School of Medicine, National University of Singapore, Blk MD10, 4 Medical Drive, Singapore 117597, Singapore

**Keywords:** Toll-like receptor 4, Microglia, NF-κB, Hypoxia-inducible factor-1α, Hypoxia, Inflammation

## Abstract

**Background:**

Hypoxia induces microglial activation which causes damage to the developing brain. Microglia derived inflammatory mediators may contribute to this process. Toll-like receptor 4 (TLR4) has been reported to induce microglial activation and cytokines production in brain injuries; however, its role in hypoxic injury remains uncertain. We investigate here TLR4 expression and its roles in neuroinflammation in neonatal rats following hypoxic injury.

**Methods:**

One day old Wistar rats were subjected to hypoxia for 2 h. Primary cultured microglia and BV-2 cells were subjected to hypoxia for different durations. TLR4 expression in microglia was determined by RT-PCR, western blot and immunofluorescence staining. Small interfering RNA (siRNA) transfection and antibody neutralization were employed to downregulate TLR4 in BV-2 and primary culture. mRNA and protein expression of tumor necrosis factor-alpha (TNF-α), interleukin-1 beta (IL-1β) and inducible nitric oxide synthase (iNOS) was assessed. Reactive oxygen species (ROS), nitric oxide (NO) and NF-κB levels were determined by flow cytometry, colorimetric and ELISA assays respectively. Hypoxia-inducible factor-1 alpha (HIF-1α) mRNA and protein expression was quantified and where necessary, the protein expression was depleted by antibody neutralization. *In vivo* inhibition of TLR4 with CLI-095 injection was carried out followed by investigation of inflammatory mediators expression via double immunofluorescence staining.

**Results:**

TLR4 immunofluorescence and protein expression in the corpus callosum and cerebellum in neonatal microglia were markedly enhanced post-hypoxia. *In vitro*, TLR4 protein expression was significantly increased in both primary microglia and BV-2 cells post-hypoxia. TLR4 neutralization in primary cultured microglia attenuated the hypoxia-induced expression of TNF-α, IL-1β and iNOS. siRNA knockdown of TLR4 reduced hypoxia-induced upregulation of TNF-α, IL-1β, iNOS, ROS and NO in BV-2 cells. TLR4 downregulation-mediated inhibition of inflammatory cytokines in primary microglia and BV-2 cells was accompanied by the suppression of NF-κB activation. Furthermore, HIF-1α antibody neutralization attenuated the increase of TLR4 expression in hypoxic BV-2 cells. TLR4 inhibition *in vivo* attenuated the immunoexpression of TNF-α, IL-1β and iNOS on microglia post-hypoxia.

**Conclusion:**

Activated microglia TLR4 expression mediated neuroinflammation via a NF-κB signaling pathway in response to hypoxia. Hence, microglia TLR4 presents as a potential therapeutic target for neonatal hypoxia brain injuries.

## Background

The developing brain is highly vulnerable to oxygen deprivation or hypoxia [1,2]. Risk factors including placental insufficiency, decreased utero-placental blood flow, as well as neonatal pulmonary and/or cardiac dysfunction can compromise neonatal oxygenation, thus affecting the development and growth of the brain [3,4]. Neuroinflammation, characterized by microglial activation, has been reported to play an important role in the hypoxic injuries in the neonatal brain [5,6]. A large number of a nascent form of microglia, known as the amoeboid microglial cells (AMCs), preponderate in the corpus callosum as well as the cerebellum of the developing brain [1]. Hypoxia-induced activation of AMCs is known to result in the production of excessive amounts of inflammatory cytokines, such as, TNF-α and IL-1β, along with nitric oxide (NO) and reactive oxygen species (ROS). Collectively, they cause oligodendrocyte death and axonal degeneration, as well as disruption of the immature blood–brain-barrier (BBB) in the periventricular white matter (PWM), leading to neonatal mortality and long-term neurodevelopmental deficits [1,6-8]. A similar phenomenon is observed in the hypoxic developing cerebellum in which activated AMCs have been shown to induce Purkinje neuronal death through production of TNF-α and IL-1β [9]. However, the mechanism via which hypoxia induces microglial activation remains to be fully explored. Hence, determination of the various mechanisms controlling microglial activation will play an important part in the suppression of neuroinflammation.

Toll-like receptors (TLRs) are first-line molecules for initiating innate immune responses. Among more than ten mammalian TLRs identified [5], TLR4 has been shown to be expressed on microglia and mediates neuroinflammatory diseases [10]. Numerous studies have demonstrated TLR4-dependent activation of microglia in neurodegenerative diseases and trauma in the central nervous system (CNS), such as Alzheimer’s disease (AD) and Parkinson’ s disease (PD) [11,12], as well as brain injury induced by ethanol [13]. Besides the above, TLR4 is also reported to be involved in hypoxia-related diseases. It has been reported recently that TLR4 is involved in brain damage and inflammation after stroke and spinal cord injury in adult mice or rats [14,15]. In fact, increased expression of TLR4 after hypoxic treatment in microglia has also been reported *in vitro*[16]; however, the expression and putative roles of TLR4 in microglia of neonatal rats following hypoxic injury have remained elusive.

In light of the critical role of TLR4 in neuroinflammation and hypoxic-ischemic-related diseases, the current study was undertaken to determine the expression, putative roles and mechanism of TLR4 in the microglia of hypoxic neonatal rats both *in vivo* and *in vitro*. Considering the involvement of hypoxia-inducible factor-1 alpha (HIF-1α) in the induction of TLR4 expression in tissue macrophages exposed to hypoxic stress [17], we sought to determine its role in TLR4 expression in hypoxic microglia. We report here that TLR4 participated in microglial activation in the hypoxic developing brain and microglia. TLR4 expression was constitutively expressed in microglia distributed in the corpus callosum and cerebellum and was noticeably increased in the brain of hypoxic pup rats. The increase in TLR4 expression in hypoxic microglia was dependent on HIF-1α, and TLR4 was found to mediate the release of pro-inflammatory mediators through the nuclear factor kappa-light-chain-enhancer of activated B cells (NF-κB) pathway. All these could collectively contribute to neonatal brain damage resulting from hypoxic exposure. Hence, regulation of TLR4 expression in microglia may therefore present as a novel therapeutic target for the treatment of various pathological states that involve hypoxia in the CNS.

## Methods

### Animals and hypoxia treatment

One-day-old Wistar rats (n = 58) were exposed to hypoxia by placing them in a chamber (Model MCO 18 M; SanyoBiomedical Electrical Co, Tokyo, Japan) filled with a gas mixture of 5% O_2_ and 95% N_2_ for 2 h. The rats were then allowed to recover under normoxic conditions for 3 and 24 h, and 3, 7 and 14 days before sacrifice; another group of 58 rats kept outside the chamber were used as age-matched controls. In addition, 3-day-old neonatal rats (n = 48) were used for the preparation of primary culture of microglia. All experiments were carried out in accordance with the National Institute of Health Guide for the Care and Use of Laboratory Animals (NIH Publications number 80–23). The project was approved by the Institutional Animal Care and Use Committee, National University of Singapore (IACUC number 095/08(A2)11). All efforts were made to reduce the number of rats used and their suffering.

### TLR4 inhibitor administration

To assess the effect of TLR4 on inflammation in neonatal brain following hypoxic injury, postnatal rats were given a singe intraperitoneal injection of TLR4-specific inhibitor CLI-095 (Invivogen, San Diego, USA, catalogue number tlrl-cli95) dissolved in dimethyl sulfoxide (DMSO) (0.5 mg/kg body weight) and grouped as follows: normal control rats, hypoxia rats, rats + DMSO, hypoxia + DMSO, rats + CLI-095, hypoxia + CLI-095. Each rat received a single injection of vehicle or inhibitor 1 h before exposure to hypoxia (n = 3 rats at each time interval for each group). A total of 38 rats were used for the drug administration and the control. As there was no noticeable change in microglial activation after DMSO injection, only results from the control, hypoxia and hypoxia + CLI-095 groups are presented.

### Primary culture and hypoxia treatment of microglial cells

Preliminary examination by immunofluorescence labeling showed an apparent increase in TLR4 expression in the corpus callosum and cerebellum. In view of this, primary culture of microglia from these brain areas was prepared for *in vitro* investigations. Glial cells were isolated from the cerebrum and cerebellum of rat pups (3-day-old) and were placed in a 75 cm^2^ flask at a density of 1.2 × 10^6^ cells/ml of DMEM (Sigma-Aldrich, St Louis, MO, USA) supplemented with 10% fetal calf serum (Hyclone, Thermo Scientific, Waltham, MA, USA), non-essential amino acids, and insulin. The flasks were then placed in a 5% CO_2_ incubator at 37°C. The medium was changed every 48 h. Once confluent (12 to 14 days), microglia were isolated from the mixed glial population by a method previously described [18]. The purity of microglia was assessed by immunocytochemical labeling using lectin from tomato (*Lycopersicon esculentum*) (1:100, Sigma, MO, USA, catalogue number L-0401), a marker of microglia. Microglial cultures with more than 96% purity were used for the study. For immunostaining (as described below) 2.5 × 10^5^ cells/well were plated in poly-L-lysine coated coverslips placed in 24-well plates. For hypoxia treatment, the culture medium was changed to fresh medium for routine culture before the cells were exposed to hypoxia by placing them in a chamber filled with a gas mixture of 3% O_2_ + 5% CO_2_ + 92% N_2_ for 24 h.

### TLR4 neutralization in primary microglia

Primary culture microglia were plated in 24-well plates with a coverslip, at a density of 2.5 × 10^5^ cells/well and divided into four groups: group I was exposed to hypoxia for 24 h; group II was treated with TLR4 neutralization antibody (10 μg/ml, a non-toxic concentration) (Santa Cruz Biotechnology, Santa Cruz, CA, USA, catalogue number sc-10741) for 1 h and immediately challenged with hypoxia for 24 h; group III was treated with TLR4 neutralization antibody for 25 h in normoxic conditions; group IV was incubated with normal complete medium and used as a control. After the various treatments, the cells were used for immunofluorescence staining.

### Double immunofluorescence labeling in postnatal rats and primary culture microglia

Double immunofluorescence was carried out in the corpus callosum and cerebellum of rats at 3 days after hypoxic exposure (n = 5) and their corresponding controls (n = 5) to confirm the expression of TLR4 in microglia. Rats were anesthetized in 6% sodium pentobarbital and perfused with a fixative containing 2% paraformaldehyde in 0.1 M phosphate buffer, pH 7.4. The brains were removed and placed in the same fixative for 4 h, after which they were kept at 4°C overnight in 0.1 M phosphate buffer containing 15% sucrose. Sections (40 μm thick) of the corpus callosum and cerebellum were cut using a cryostat (Leica Microsystems Nussloch GmbH, Nussloch, Germany). The sections were washed with PBS, blocked with 5% normal serum for 1 h, and incubated in anti-rabbit TLR4 polyclonal antibody (dilution 1:100; Santa Cruz Biotechnology, catalogue number sc-10741) overnight at room temperature. After incubation, Cy3-conjugated secondary antibody was added and incubated at room temperature for 1 h. The sections were again incubated with the FITC-conjugated lectin from tomato (*Lycopersicon esculentum*) (1:100). Double immunofluorescence staining was also carried out with TLR4 and OX42 (1:100, Chemicon, International, Temecula, CA, catalogue number CBL1512) for the corpus callosum. The sections were then washed in PBS and mounted using a fluorescent mounting medium (Dako, Oregon City, USA, catalogue number S3023). Cellular localization was then examined under a confocal microscope (FV1000; Olympus, Tokyo, Japan) with the same exposure settings for each comparison group. Double immunofluorescence was also carried out in hypoxic rats to investigate the changes of TNF-α, IL-1β and iNOS expression after injection of TLR4 inhibitor. Double immunofluorescence staining of iNOS (anti-mouse 1:100, BD Pharmingen, San Jose, CA USA, catalogue number 610432) expression at 3 h, as well as that of TNF-α (1:100; anti-rabbit polyclonal, Millipore Bioscience Research Reagents, Billerica, MA, USA, catalogue number AB1837P) and IL-1β (1:100, anti-rabbit polyclonal, Millipore Bioscience Research Reagents, catalogue number AB1832P) at 3 d after hypoxia in microglia (lectin labeled) in CLI-095-injected rats and the corresponding controls were processed as described above. For double immunofluorescence staining in the primary microglia, the cells were fixed with 4% paraformaldehyde for 20 minutes and separately incubated with anti-rabbit TLR4, anti-rabbit TNF-α, anti-rabbit IL-1β, anti-mouse iNOS and anti-rabbit NF-κB/p65 (1:100, Santa Cruz Biotechnology, catalogue number sc-109) and were processed with the immunofluorescence staining as described above, then the sections were mounted using a fluorescent mounting medium (Sigma, catalogue number F6057).

### BV-2 cell culture and hypoxia treatment

BV-2 cells were used for *in vitro* study because our recent studies [19,20] have shown that this microglial cell line responds swiftly to hypoxia exposure. This was confirmed in this study, in which expression of HIF-1α was readily detected in hypoxic BV-2 cells, and the induced HIF-1α expression was acute in onset. BV-2 cells were cultured at 37°C in growth medium containing DMEM supplemented with 2% fetal bovine serum (FBS) (Invitrogen, Carlsbad, CA, USA), and 1% antibiotic in a humidified incubator containing 5% CO_2,_ and 95% air. The culture medium was changed to fresh medium for routine culture before the cells were exposed to hypoxia by placing them in a chamber filled with a gas mixture of 3% O_2_ + 5% CO_2_ + 92% N_2_ for 2, 4, 6, 8, 12 and 24 h.

### HIF-1α neutralization in BV-2 microglia

BV-2 microglia were plated in 24-well plates with coverslips at a density of 1.5 × 10^5^ cells/well and divided into four groups: group I was subjected to hypoxia for 8 h; group II was treated with HIF-1α antibody at (10 μg/ml, a non-toxic concentration) (Chemicon, catalogue number 400080) for 1 h and immediately challenged with hypoxia for 8 h; group III was treated with HIF-1α antibody for 9 h in normoxic conditions; group IV was incubated with normal growing medium and was used as a control. After various treatments, the cells were used for immunofluorescence staining. For western blot analysis, BV-2 cells were plated in 6-well plates following the above treatments.

### Silencing of TLR4 with small interfering RNA (siRNA)

TLR4 expression was silenced using TLR4 small interfering RNA (siRNA) (Ambion, Foster City, CA, USA, catalogue number s75207) according to the manufacturer’s instructions. Non-treated BV-2 cells and BV-2 cells transfected with nonspecific scramble siRNA that does not target any mouse genes (Control siRNA) were used as controls. The reverse transfection method was adopted for silencing. Briefly, after subculture, BV-2 cells were resuspended in Optimem (GIBCO, Invitrogen, catalogue number 31985070) and plated in 6-well plates at a density of 3 × 10^5^ cells/ml. This was followed by adding 500 μl Optimem with 10 μl siRNA and 4 μl lipofectamine dropwise in the above well. The cells were incubated with the siRNA mix for 8 h and then the medium was replaced with DMEM with 2% FBS without antibiotics and incubated for another 16 h for RNA extraction to check the knockdown efficiency by reverse transcription (RT)-PCR. The microglia were subjected to hypoxia for 8 h at 40 h after transfection. After that, cells were either fixed for immunofluorescence staining, or protein was extracted for western blotting as below. For cell viability analysis, reverse transfection was carried out in a 24-well plate. At 40 h after transfection, both the transfected and non-transfected BV-2 cells were subjected to hypoxia for 8 h.

### Cell viability analysis of BV-2 cells

The effect of hypoxia and siRNA transfection on the viability of BV-2 cells was evaluated by CellTiter 96® AQueous One Solution Cell Proliferation Assay kit (Promega, Fitchburg, WI, USA, catalogue number G3580). The cell viability of the non-treated BV-2 cells, control siRNA transfected BV-2 cells, TLR4 siRNA transfected BV-2 cells and the corresponding cells subjected to hypoxia for 8 h was measured. We added 3-(4,5-dimethylthiazol-2-yl)-5-(3-carboxymethoxyphenyl)-2-(4-sulfophenyl)-2 h-tetrazolium, inner salt (MTS) reagent into each well (20 μl/well) followed by incubation for 4 h at 37°C in a humidified atmosphere of 5% CO_2_ and 95% air, before the absorbance at 490 nm was measured using a microplate reader (GENIOS, Tecan, Switzerland). Cell viability was expressed as a percentage of control BV-2 cells.

### Immunofluorescence staining in BV-2 cells

BV-2 cells were fixed with 4% paraformaldehyde in 0.1 M PBS for 15 minutes. Following rinsing with PBS, the coverslips with adherent cells were used for immunofluorescence staining. In every group, BV-2 cells were incubated, with either anti-rabbit TLR4 (1:100), anti-mouse HIF-1α (1:100), anti-rabbit TNF-α (1:100; Chemicon, Temecula, CA, USA, catalogue number AB2148P), anti-rabbit IL-1β (1:100; Chemicon, catalogue number AB1413), anti-mouse iNOS (1:100) or anti-rabbit NF-κB/p65 (1:100) overnight at room temperature. Subsequently, the cells were incubated in FITC/Cy3-conjugated secondary antibodies for 1 h at room temperature. After washing, the coverslips were mounted using a fluorescent mounting medium with 4^′^,6-diamidino-2-phenylindole (DAPI). All images were captured using a confocal microscope (Fluoview1000, Olympus, Tokyo, Japan).

### Real time RT-PCR

Total RNA was extracted from all of the cells using the RNeasy Mini kit (Qiagen, Valencia, CA, USA). RT reactions were performed using the RT system kit (Promega, Singapore). The resultant cDNA was diluted 10 times in double distilled H_2_O and kept at −20°C for RT-PCR analysis. Primer pairs for *HIF-1α*, *TNF-α*, *IL-1β*, *iNOS* and *β-actin* were designed using the primer design program (Primer 3 software version 1.0). The primer sequences for the genes and their corresponding amplicon size are listed in Table 1. RT-PCR was performed using a LightCycler (Roche Diagnostics, Indianapolis, IN, USA), and individual RT-PCRs were carried out in glass Light Cycler capillaries (Roche Diagnostics) according to the manufacturer’s instructions. The RT-PCRs were carried out in a 10-μl final volume containing the following: 5 μl 2xSYBR Green I master mix (Qiagen); 1 μl of 5 μM forward primer and 1 μl of 5 μM reverse primer; and 3 μl of diluted cDNA. After an initial denaturation step at 95°C for 15 minutes, temperature cycling was initiated. Each cycle consisted of denaturation at 94°C for 15 sec, annealing at 60°C for 25 sec, and elongation at 72°C for 20 sec. In total, 55 cycles were performed. Mouse β-actin was amplified as the control for normalizing the quantities of transcripts of each of the genes mentioned above. The differences in expression for *HIF-1α*, *TNF-α*, *IL-1β* and *iNOS* between the control and treated cells were calculated by normalizing with the *β-actin* gene expression according to the following formula [21]:

$$-\text{Fold}\;\text{change}=2^{-\left[\mathrm{Ct}\left(\text{control}\right)\;\text{gene}\times -\mathrm{Ct}\left(\text{control}\right)\;\text{actin}\right]-\left[\mathrm{Ct}\left(\text{activated}\right)\;\text{gene}\times -\;\mathrm{Ct}\left(\text{activated}\right)\;\text{actin}\right]}.$$

**Table 1:** Sequence of specific primers used for quantitative real-time PCR

**Gene**		**Sequence**
*TLR4*	Forward	ctacctggaatgggaggaca
	Reverse	cttagcagccatgtgttcca
*HIF-1α*	Forward	gcagcaggaattggaacatt
	Reverse	gcatgctaaatcggagggta
*TNF-α*	Forward	cgtcagccgatttgctatct
	Reverse	cggactccgcaaagtctaag
*IL-1β*	Forward	gcccatcctctgtgactcat
	Reverse	aggccacaggtattttgtcg
*iNOS*	Forward	gcttgtctctgggtcctctg
	Reverse	ctcactgggacagcacagaa
*NF-κB*	Forward	gcgtacacattctggggagt
	Reverse	ccgaagcaggagctatcaac

TLR4, Toll-like receptor 4; HIF-1α, Hypoxia-inducible factor-1 alpha; iNOS, Inducible nitric oxide synthase; NF-κB, Nuclear factor kappa-light-chain-enhancer of activated B cells.

### Western blotting analysis

Culture medium was removed from the culture plate, and cells were washed twice with ice-cold PBS. Cells were lysed with lysis buffer, mechanically scraped off with a rubber scraper and centrifuged at 13,000 rpm for 25 minutes. Protein concentration of samples was then determined by using a protein assay kit (Bio-Rad, Hercules, CA, USA, catalogue number 500–0002). Next, 20 μg of the protein sample was loaded and separated on 10% sodium dodecyl sulfate-polyacrylamide gels. The proteins embedded in the gel were then transferred to polyvinylidene difluoride membranes using a semidry electrophoretic transfer cell (Bio-Rad). The membranes were washed with TBS-0.1% Tween buffer and then incubated with 5% nonfat dry skim milk for 30 minutes at room temperature. Next, they were incubated with anti-mouse TLR4 (1:1000; Santa Cruz Biotechnology, catalogue number sc-293072), anti-mouse HIF-1α (1:1000), anti-rabbit TNF-α (1:1000), anti-rabbit IL-1β (1:1000), anti-rabbit NF-κB/p65 (1:1500), and anti-mouse β-actin (dilution 1:10,000; Sigma-Aldrich, catalogue number A5441) overnight on a shaker at 4°C. After three washes with TBS-0.1% Tween, the membranes were incubated with horseradish peroxidase-conjugated secondary antibody for 1 h. The proteins were detected with a chemiluminescence detection system according to the manufacturer’s instruction (Supersignal West Pico Horseradish Peroxidase Detection Kit; Pierce Biotechnology, Rockford, IL, USA, catalogue number 34077) and developed on the film. The band intensity was quantified in Image J software (National Institutes of Health, NIH, USA). All experiments were repeated at least in triplicate.

### Assay of TNF-α and IL-1β concentration in primary microglia by ELISA

The levels of TNF-α and IL-1β in the supernatant of primary cultured microglia after hypoxia and TLR4 neutralization were determined with TNF-α ELISA kit (IBL, Hamburg, Germany, catalogue number BE45471) and IL-1β ELISA kit (IBL, catalogue number 27193). The ELISA measurements were performed according to the manufacturer’s instructions.

### Measurement of reactive oxygen species by flow cytometry

Intracelluar ROS production in BV-2 cells of different groups was evaluated by detecting the fluorescence intensity of 2^′^, 7^′^-dichlorofluorescene, the oxidized product of the fluoroprobe 5-(and 6)-chloromethyl-2^′^, 7^′^-dichlorodihydrofluorescein diacetate (CM-H2DCFDA, Molecular Probes, Invitrogen, catalogue number C6827) according to the manufacturer’s instruction. The amount of ROS production was considered to be directly proportional to fluorescence intensity given as cell counts and fluorescence intensity at the y-axis in the flow cytometry.

### Nitric oxide concentration measurement

BV-2 cells were treated as described above and the supernatant was collected. NO concentration was measured by NO colorimetric BioAssay™ Kit (US Biological, Swampscott, MA, USA, catalogue number K262-200), according to the manufacturer’s instruction.

### Phosphorylated-NF-κB p65 protein level analysis

After siRNA transfection, the cell pellets were collected and then the total protein in control and treated BV-2 cells was extracted. The protein concentration was measured by Pierce BCA protein Assay Kit (Pierce Biotechnology). Phospho-NF-κB/p65 protein level analysis was carried out using PathScan Phospho-NF-κB/p65 (Ser536) Sandwich ELISA Kit (Cell signaling, Danvers, MA, USA, catalogue number 7173) according to the manufacturer’s instruction.

### Statistical analyses

The data were presented as mean ± SD. The statistical significance of differences between control, hypoxic and treatment groups was calculated using Student’s *t*-test and one-way analysis of variance (ANOVA). Statistical significance was determined by *P <0.05 and **P <0.01.

## Results

### TLR4 expression is increased in cerebral and cerebellar microglia after hypoxia exposure in neonatal rats

**Figure 1:**
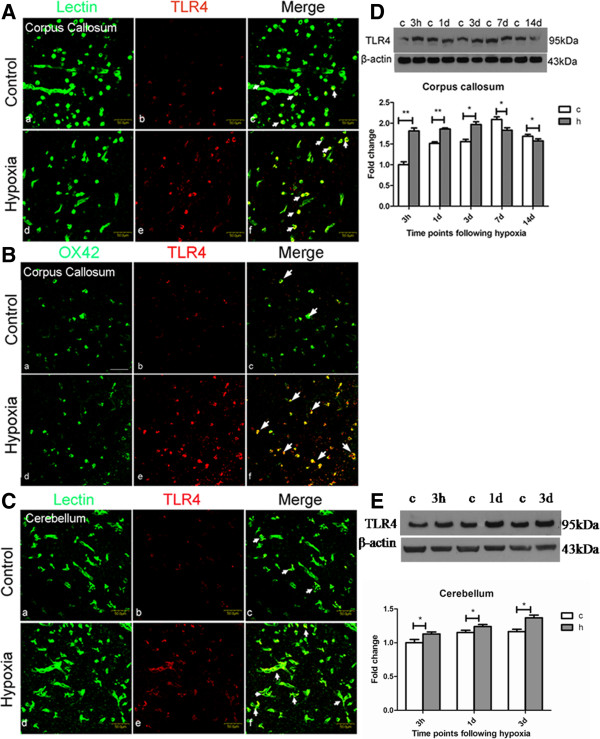
**Toll-like receptor 4 (TLR4) immunofluorescence and protein expression was increased in the corpus callosum and cerebellum in neonatal rats following hypoxic exposure.** Confocal images showing the distribution of lectin/OX42 (green) and TLR4 (red) immunoreactive cells in the corpus callosum and cerebellum at 3 days after hypoxic exposure and the corresponding control (**A-C**). Colocalized expression of TLR4 and lectin/OX42 immunoreactive cells (arrows) in corpus callosum (Ac, Af; Bc, Bf) and cerebellum (Cc, Cf) can be seen. Note the upregulated expression of TLR4 in some lectin/OX42-positive microglial cells after hypoxia (Af, Bf, Cf). Western blotting of TLR4 protein expression in the corpus callosum (3 h, and 1, 3, 7 and 14 days) and cerebellum (3 h, and 1 and 3 days) of rats after hypoxic exposure and their corresponding controls is shown. The upper panels show specific bands of TLR4 (95 kDa) and β-actin (43 kDa). The lower panels are bar graphs showing significant changes in the optical density following hypoxic exposure (h) (normalized with β-actin, shown as fold change of control in 3 h (c)). TLR4 protein expression in the corpus callosum is significantly increased at 3 h, and 1 and 3 days after hypoxic exposure when compared with the matching control. At 7 and 14 days, TLR4 protein expression level was declined in comparison with the comparing control (**D**). TLR4 protein expression level in the cerebellum also increased significantly following hypoxia at 3 h, and 1 and 3 days when compared with the matching control (**E**). Significant differences in protein levels between hypoxic and control rats are expressed as *P <0.05 and **P <0.01. Fold-change values are calculated from triplicates and represented as mean ± SD. Scale bars in A**-**C = 50 μm. TLR4, Toll-like receptor 4.

**Figure 2:**
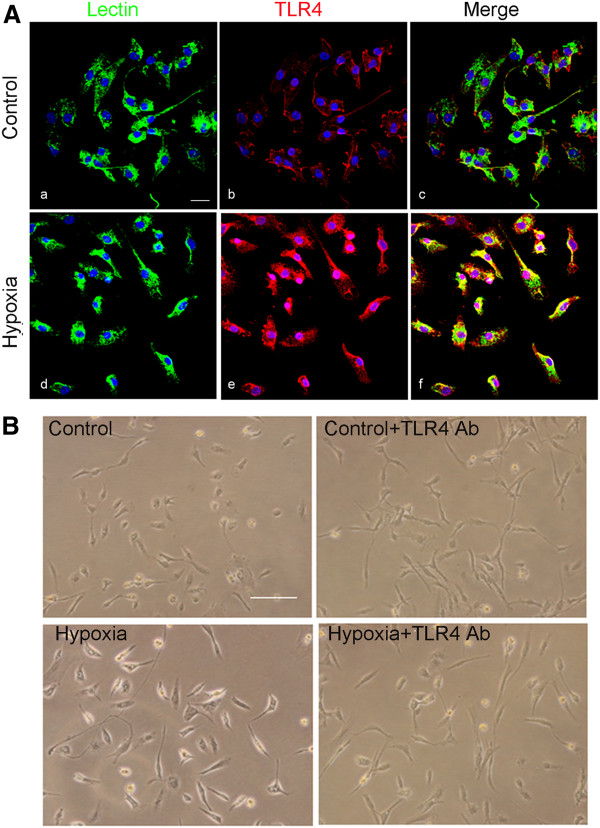
**Toll-like receptor 4 (TLR4) expression was increased in primary cultured microglia following hypoxia.** (**A**) Confocal images showing the expression of TLR4 (Ab, Ae; red) in primary cultured microglia labeled with lectin (Aa, Ad; green) in both control and hypoxia for 24 h. TLR4 immunoflurosence intensity is markedly enhanced after hypoxia exposure (Af) in comparison with the control (Ac). Nuclei are stained with DAPI (blue). (**B**) TLR4 was neutralized with its antibody in control and hypoxia conditions. In control microglia, microglia + TLR4 Ab, microglia + hypoxia and microglia + hypoxia + TLR4 Ab, there is no noticeable difference in cell morphology between different groups under the phase-contrast microscope. Scale bars = 20 μm (**A**) and 100 μm (**B**).

To assess the role of TLR4 in microglia in the developing brain following a hypoxic injury, we first profiled the change in TLR4 expression in microglia in the corpus callosum and cerebellum, as well as cultured microglia subjected to hypoxia. *In vivo*, most TLR4 expression was colocalized in the lectin/OX42-labeled microglia in the corpus callosum as well as in the white matter of the cerebellum (Figure 1). In the corpus callosum, weak TLR4 expression was expressed in sporadic microglia but was increased in microglia 3 days after hypoxia (Figure 1A and B). In the cerebellum, TLR4 immunoexpression, which was weak in the intensity of microglia in control rats, was also enhanced at 3 days after hypoxic exposure (Figure 1C). Western blot analysis of protein from the corpus callosum showed that TLR4 protein expression level was increased at 3 h after hypoxic exposure when compared with the matching control. A similar phenomenon was observed in rats killed at 1 and 3 days after hypoxia (Figure 1D). Likewise, TLR4 protein level in the cerebellum also increased significantly the first 3 days after hypoxia compared to control by western blot analysis (Figure 1E). Consistent with results *in vivo*, changes in TLR4 immunoexpression were also observed in the primary cultured microglia, in which TLR4 immunofluorescence intensity was markedly enhanced versus controls when the cells were subjected to hypoxia for 24 h (Figure 2A). It is noteworthy that microglial external morphology and cell density remained relatively unaltered after 24 h of hypoxia (Figure 2B).

### TLR4 blocking in primary microglia reduced production of inflammatory mediators

**Figure 3:**
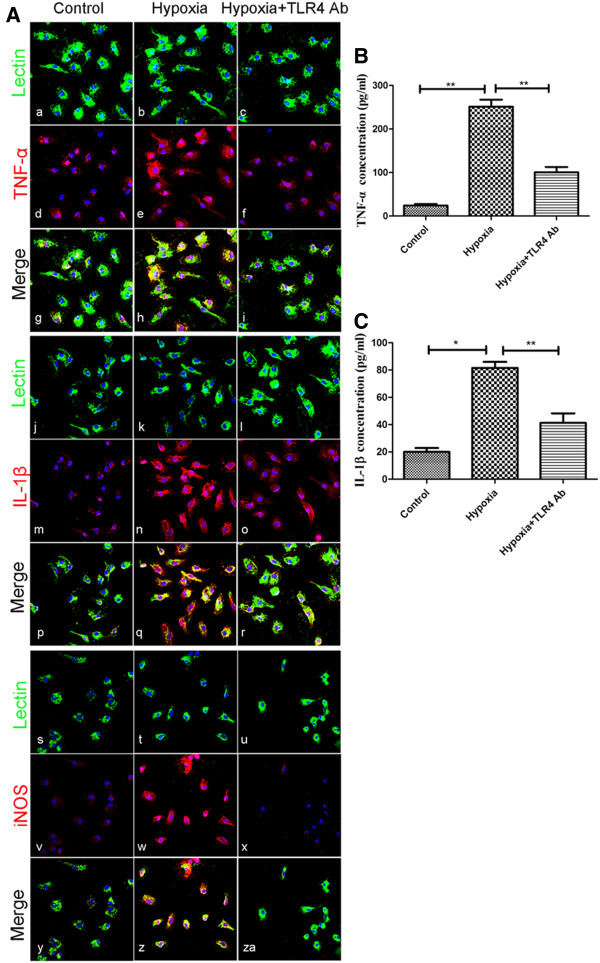
**Neutralization of Toll-like receptor 4 (TLR4) with its antibody attenuated the hypoxia-induced expression of TNF-α, IL-1β, and inducible nitric oxide synthase (iNOS) in primary cultured microglia.** TLR4 was neutralized with its antibody to assess its role in production of inflammatory factors. (**A**) Confocal images showing the expression of TNF-α (d-f), IL-1β (m-o) and iNOS (v-x) in primary microglia labeled with lectin (a-c, j-l, s-u; green) in different groups. Very weak TNF-α, or almost undetectable IL-1β and iNOS immunoexpression is detected in the control microglia (g, p, y). The immunoflurescence intensity is enhanced in microglia subjected to hypoxia exposure (h, q, z). This increased immunofluorescence intensity is attenuated in microglia exposed to hypoxia but pretreated with TLR4 Ab (i, r, za). TNF-α (**B**) and IL-1β (**C**) concentration in the supernatant of microglia in different groups was investigated with ELISA. Release of TNF-α and IL-1β is significantly increased after hypoxia in comparison to the control levels. The hypoxia-induced increase in TNF-α and IL-1β release was suppressed when the microglia were neutralized with TLR4 antibody. Significant differences in protein levels between different groups are indicated as *P <0.05 and **P <0.01. The values represent the mean ± SD in triplicate. Scale bars in **A** = 20 μm.

To investigate the role of TLR4 in the activated microglia, we blocked TLR4 using its antibody before hypoxic exposure and then examined the expression of inflammatory mediators. Treatment of primary microglia with TLR4 antibody did not affect the cell morphology and cell density (Figure 2B). TNF-α, IL-1β and iNOS immunofluorescence in microglia was noticeably enhanced after hypoxic exposure but was attenuated in hypoxic microglia pretreated with TLR4 antibody (Figure 3A). To further confirm the changes in TNF-α and IL-1β, quantitative anlysis of TNF-α and IL-1β in the supernatant of cultured microglia was performed by ELISA. In parallel to the changes in the protein expression revealed by immunofluorescence staining, both the secretion of TNF-α and IL-1β, especially the former, was increased after hypoxic treatment and the increase was markedly suppressed by TLR4 neutralization (Figure 3B and C).

### Hypoxic exposure up-regulated TLR4 expression in BV-2 cells

**Figure 4:**
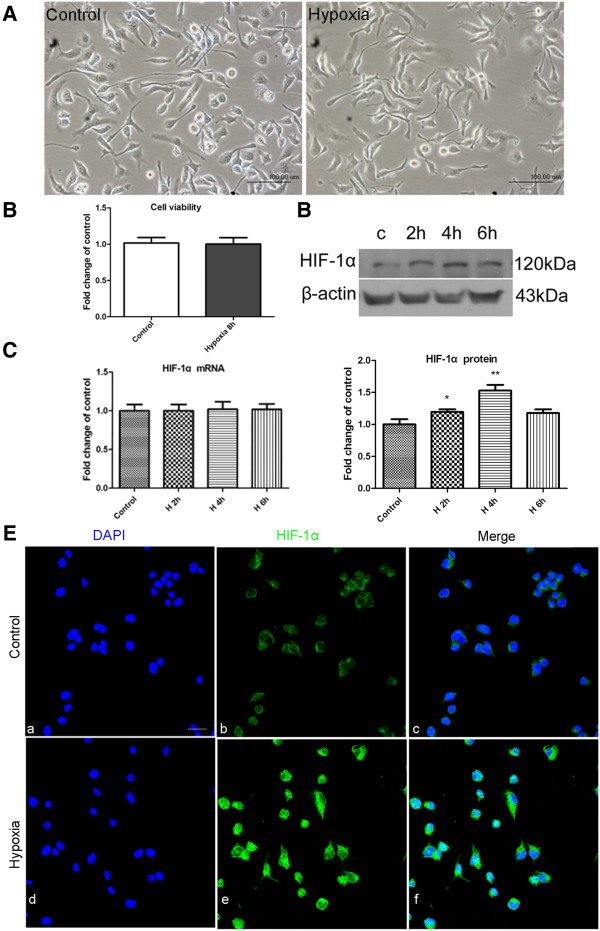
**Hypoxia-inducible factor-1 alpha (HIF-1α) expression in hypoxic BV-2 cells.** (**A**) The external cell morphology appears relatively unchanged in BV-2 cells exposed to hypoxia for 8 h when compared with the control. (**B**) Bar graph shows the viability of BV-2 cells after hypoxia compared to control. Note that the viability of cells in the two groups is comparable. (**C**) Reverse transcription (RT)-PCR analysis of HIF-1α mRNA expression in BV-2 cells exposed to hypoxia for 2, 4, and 6 h and control (c). Note that there is no significant difference between different groups. (**D**) Western blot analysis of HIF-1α protein expression in different groups. Specific band of HIF-1α (120 kDa) and β-actin (43 kDa) is shown in the upper panel of **D**. Bar graphs in the lower panel of **D** shows significant changes in the optical density following 2 and 4 h of hypoxic exposure (normalized with β-actin, shown as fold change of control), notably after exposure to hypoxia for 4 h. (**E**) Confocal images showing the expression of HIF-1α in BV-2 cells and those exposed to hypoxia for 4 h. Weak HIF-1α expression is detected in the control BV-2 cells (Eb, Ec). The immunofluorescence intensity is enhanced markedly after hypoxic exposure (Ee, Ef). Significant differences in protein levels between hypoxic and control BV-2 cells are indicated as *P <0.05 and **P <0.01. The values represent the mean ± SD in triplicate. Scale bars = 100 μm (**A**) and 20 μm (**E**).

**Figure 5:**
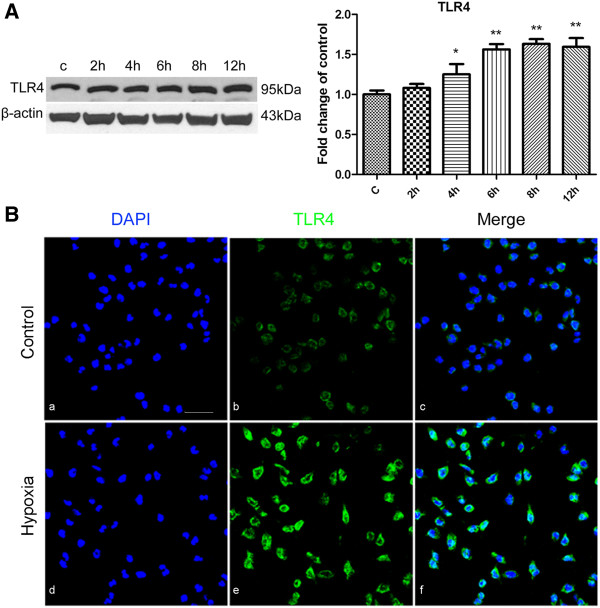
**Toll-like receptor 4 (TLR4) expression was increased in BV-2 cells following hypoxia.** (**A**) Western blotting of TLR4 protein expression in BV-2 cells exposed to hypoxia for 2, 4, 6, 8 and 12 h and control (c). The left panel shows specific bands of TLR4 (95 kDa) and β-actin (43 kDa). The right panel is a bar graph showing significant changes in the optical density following hypoxic exposure (normalized with β-actin, shown as fold change of control). Note significant increase in TLR4 expression after hypoxic treatment of varying durations in BV-2 cells, especially at 8 h. (**B**) Confocal images showing TLR4 expression in control BV-2 cells and those exposed to hypoxia for 8 h. Weak TLR4 expression is detected in the control BV-2 cells (Bb, Bc), with enhanced immunofluorescence intensity after 8 h of hypoxic exposure (Be, Bf). Significant differences between control and hypoxic BV-2 cells are expressed as *P <0.05 and **P <0.01. The values represent the mean ± SD in triplicate. Scale bar in **B** = 20 μm.

The BV-2 microglial cell line was used for the investigation of TLR4 function in hypoxic microglia. The HIF-1α subunit is tightly regulated by oxygen and its upregulation is regarded as evidence of the hypoxic condition. To confirm that the hypoxic effect on BV-2 cells was successfully achieved, HIF-1α expression was first assessed by RT-PCR and western blot analysis. The external cell morphology of BV-2 cells subjected to hypoxia for 8 h appeared relatively unchanged (Figure 4A) and the cell viability was not significantly different from the control cells (Figure 4B). By RT-PCR, HIF-1α mRNA expression level was comparable between the hypoxia and control BV-2 cells (Figure 4C). In contrast, protein expression of HIF-1α was significantly increased following 2 and 4 h hypoxic exposure, especially so with 4 h of hypoxia (*P*<0.01) (Figure 4D). Immunofluorescence staining showed that HIF-1α, which was weakly expressed in normal BV-2 cells, was markedly enhanced after 4 h of hypoxia (Figure 4E). TLR4 protein expression level in BV-2 cells was compared between the control and hypoxic groups. TLR4 was increased after 4, 6, 8 and 12 h of hypoxic exposure for which the TLR4 protein expression was most pronounced at 8 h as revealed by western blot (Figure 5A). Immunofluorescence staining confirmed that TLR4 was weakly expressed in normal BV-2 cells but its immunofluorescence intensity was increased conspicuously after 8 h of hypoxia (Figure 5B).

### Silencing of TLR4 gene in BV-2 cells

**Figure 6:**
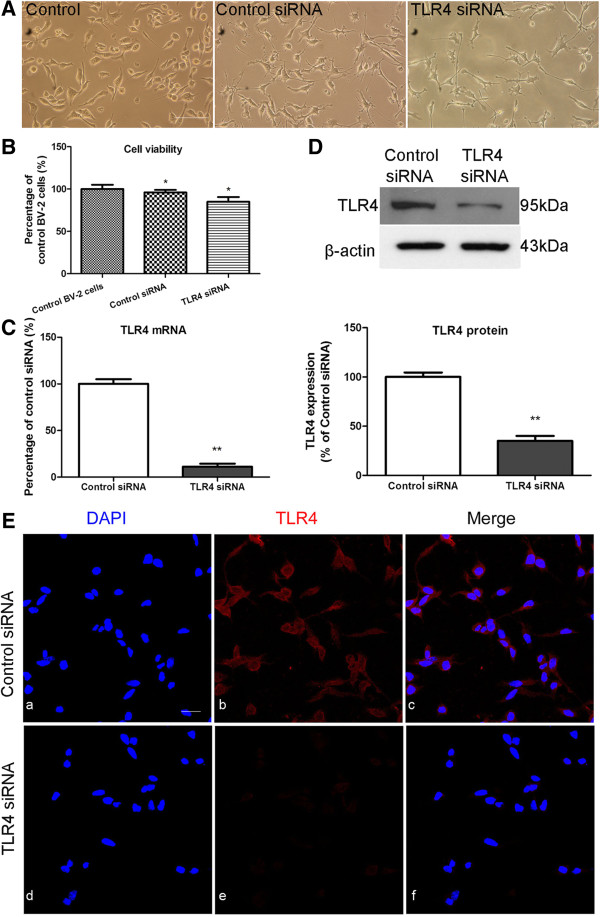
**Downregulation of Toll-like receptor 4 (TLR4) after TLR4 siRNA transfection in BV-2 cells.** The cells appear more ramified with long extending processes when transfected with either control siRNA or TLR4 siRNA, when compared with the non-transfected control cells under the phase-contrast microscope (**A**). The viability of BV-2 cells transfected with control siRNA and TLR4 siRNA is 96% and 85%, respectively, of the non-transfected control value (**B**). Silencing efficiencies were analyzed by reverse transcriptase (RT)-PCR (**C**), western blots (**D**) and immunofluorescence staining (**E**) at 24, 48 and 48 h after transfection respectively. RT-PCR analysis shows that the efficiency of siRNA-mediated suppression of TLR4 is about 88.9% compared to negative control (normalized with β-actin) (**C**). (**D**) The upper panel shows the specific band of TLR4 (95 kDa) and β-actin (43 kDa). The lower panel shows bar graphs depicting significant changes in the optical density following hypoxic exposure (given as fold change of the control siRNA transfected group). Note the remaining TLR4 protein expression in TLR4 siRNA-transfected BV-2 cells is about 38% compared to the control siRNA-transfected BV-2 cells. Immunofluorescence images show that TLR4 immunoreactivity is markedly reduced in TLR4 siRNA transfected BV-2 cells compared to negative control (**E**). *P <0.05 and **P <0.01. The values represent the mean ± SD in triplicate. Scale bars = 100 μm (**A**) and 20 μm (**E**).

To further confirm the role of TLR4 in BV-2 cells after hypoxic treatment, we used siRNA knockdown TLR4 gene in BV-2 cells. BV-2 cells were either transfected with TLR4 siRNA or control siRNA. Transfected BV-2 cells appeared more ramified when compared with the control BV-2 cells under the phase-contrast microscope (Figure 6A). Compared with the non-transfected control BV-2 cells, the cell viability was about 96% after control siRNA transfection, and 85% after TLR4 siRNA transfection (Figure 6B). TLR4 mRNA expression level in BV-2 cells was significantly decreased when transfected with TLR4 siRNA. The silencing efficiency was achieved at about 88.96% at 24 h when compared to control siRNA-transfected cells (Figure 6C). Moreover, TLR4 protein expression was reduced by about 65% in TLR4 siRNA-transfected cells compared to control siRNA-transfected cells at 48 h after transfection as revealed by western blot analysis (Figure 6D). This was further verified by confocal immunofluorescence microscopy, which showed an obvious reduction in TLR4 immunostaining in TLR4 siRNA-transfected BV-2 cells (Figure 6E).

### Knockdown of TLR4 expression in BV-2 cells inhibited production of inflammatory mediators

**Figure 7:**
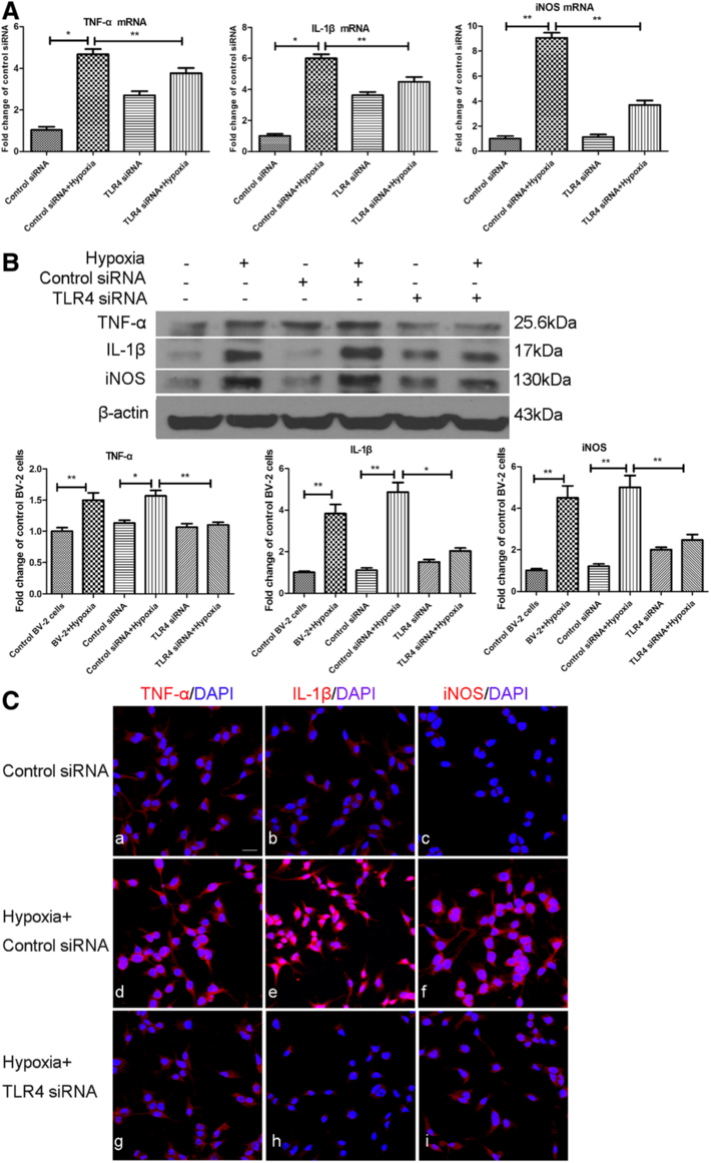
**Suppression of Toll-like receptor 4 (TLR4) with siRNA reduced the mRNA, protein expression and immunoreactivity of TNF-α, IL-1β and inducible nitric oxide synthase (iNOS) induced by hypoxic stress in BV-2 cells.** (**A**) Reverse transcriptase (RT)-PCR analysis of *TNF-α*, *IL-1β* and *iNOS* gene expression in BV-2 cells transfected with control siRNA, transfected with control siRNA + hypoxia, transfected with TLR4 siRNA and TLR4 siRNA + hypoxia. Note that TNF-α, IL-1β and iNOS mRNA expression is increased significantly by different amountsrespectively after hypoxic exposure in control siRNA-transfected BV-2 cells (TNF-α, 5-fold; IL-1β, 6-fold; iNOS, 9-fold) (normalized with β-actin), but the increase was significantly suppressed in hypoxic TLR4 siRNA-transfected BV-2 cells compared with control siRNA-transfected BV-2 cells exposed to hypoxic stress. Notably, iNOS mRNA expression is decreased by 55% of the control. (**B**) Western blot analysis of TNF-α, IL-1β and iNOS protein expression in different groups of BV-2 cells given different treatments. The upper panel shows specific bands of TNF-α (25.6 kDa), IL-1β (17 kDa), iNOS (130 kDa) and β-actin (43 kDa). The lower panel of bar graphs shows significant changes in the optical density following hypoxic exposure (given as fold change of control siRNA transfected group). TNF-α, IL-1β and iNOS protein expressions are significantly increased after hypoxic exposure in control siRNA-transfected BV-2 cells and this increase is significantly decreased after hypoxic exposure in TLR4-transfected groups. (**C**) Immunofluorescence images show that TNF-α, IL-1β and iNOS immunoreactivity is markedly reduced in TLR4 siRNA-transfected BV-2 cells subjected to hypoxic exposure compared to cells transfected with control siRNA. *P <0.05 and **P <0.01. The values represent the mean ± SD in triplicate. Scale bar in **C** = 20 μm.

**Figure 8:**
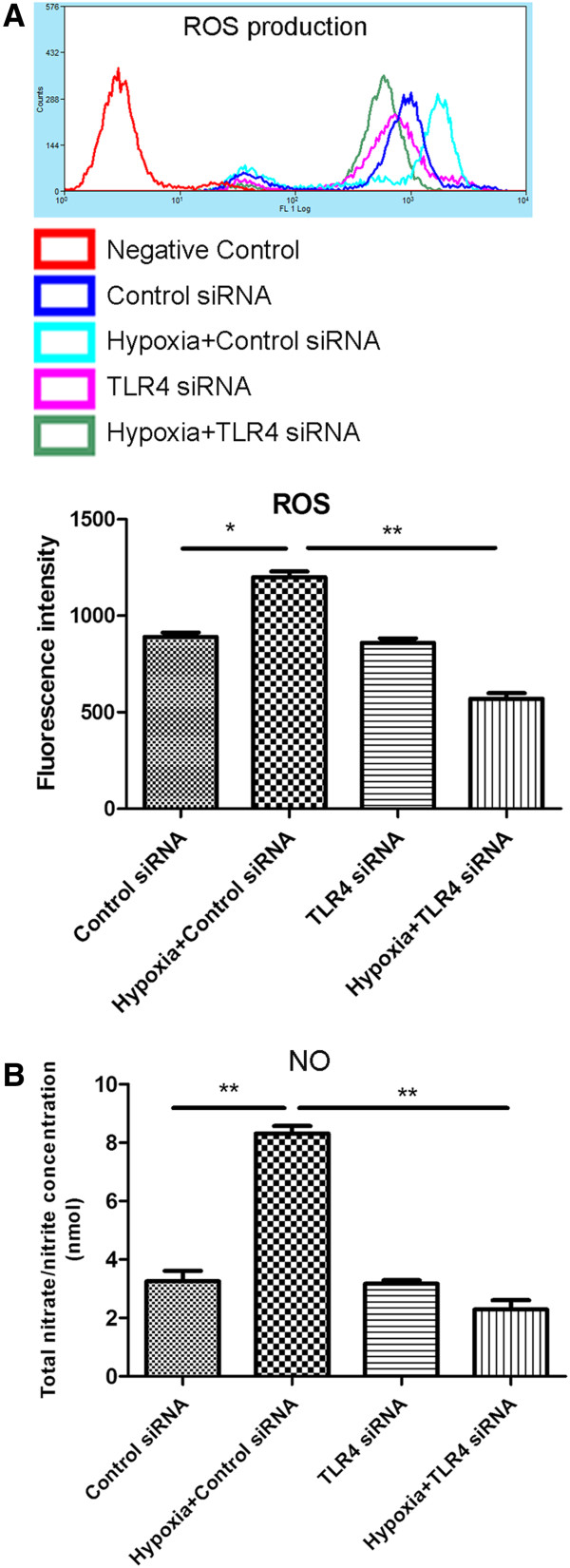
**Suppression of Toll-like receptor 4 (TLR4) with siRNA reduced the production of reactive oxygen species (ROS) and nitric oxide (NO) induced by hypoxic stress in BV-2 cells.** Intracellular ROS and production of NO in BV-2 cells transfected with control siRNA, control siRNA + hypoxia, transfected with TLR4 siRNA and TLR4 siRNA + hypoxia were checked. (**A**) The upper panel shows cell counts (y-axis) and log10 expression of fluorescence intensity (x-axis). The lower panel is a bar graph showing a significant change in the fluorescence intensity of intracellular ROS production following the above treatments. Note the ROS production, which is increased after hypoxic exposure in control siRNA-transfected BV-2 cells, is significantly decreased after hypoxic exposure in the TLR4-transfected group. (**B**) NO production in supernatant also shows a similar change as with ROS in the different groups mentioned above *P <0.05 and **P <0.01. The values represent the mean ± SD in triplicate.

Increase in mRNA expression of hypoxia-induced inflammatory mediators such as TNF-α, IL-1β and iNOS was partially inhibited in hypoxic BV-2 cells with TLR4 gene knockdown. TNF-α mRNA expression level was increased five-fold in BV-2 cells transfected with control siRNA and subjected to hypoxia for 8 h; however, when BV-2 cells transfected with TLR4 siRNA were subjected to hypoxia, the increase was inhibited significantly. A similar trend was observed in IL-1β and iNOS mRNA expression levels across the different groups. The most striking change was observed in iNOS mRNA expression, which was increased by nine times after BV-2 cells were transfected with control siRNA and subjected to hypoxia for 8 h. It was substantially decreased by about 55% in BV-2 cells transfected with TLR4 siRNA and exposed to hypoxia for the same duration (Figure 7A). In parallel with mRNA expression, western blot results demonstrated that TNF-α, IL1-β and iNOS protein expression levels were also inhibited considerably in hypoxic BV-2 cells transfected with TLR4 siRNA compared with those transfected with control siRNA (Figure 7B). Confocal immunofluorescence microscopy showed an obvious reduction in TNF-α, IL-1β and iNOS immunostaining intensity in hypoxic BV-2 cells with TLR4 knockdown compared with the hypoxic control siRNA-transfected BV-2 cells (Figure 7C). Flow cytometry results showed that intracellular ROS production, which was significantly increased in hypoxic control siRNA-transfected BV-2 cells, was suppressed significantly in hypoxic BV-2 cells with TLR4 knockdown compared with the former (Figure 8A). In addition, similar changes in NO concentration in the supernatant of different groups mentioned above were observed (Figure 8B).

### Hypoxia-induced increase in TLR4 expression in BV-2 cells was via HIF-1α

**Figure 9:**
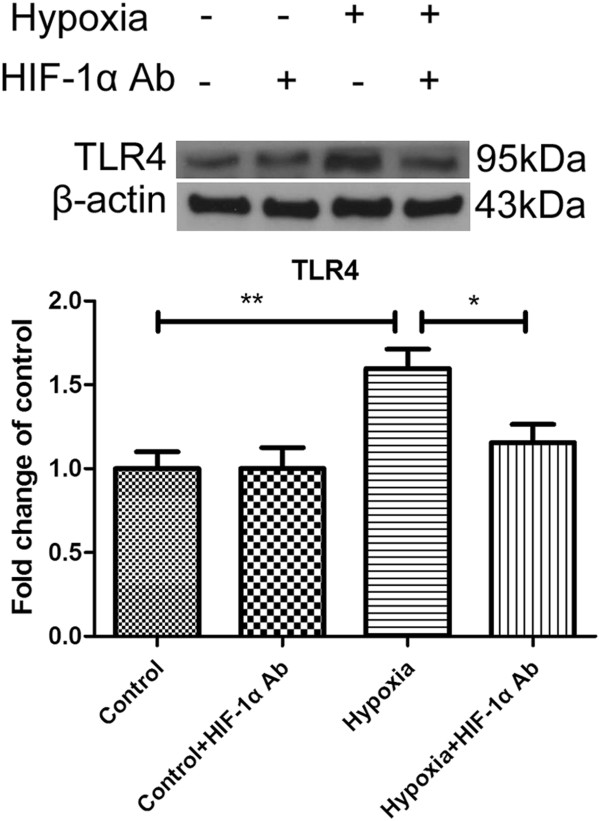
**Neutralization of Hypoxia-inducible factor-1 alpha (HIF-1α) with its antibody attenuated the hypoxia-induced expression of Toll-like receptor 4 (TLR4) in BV-2 cells.** HIF-1α was neutralized with its antibody to assess its role in TLR4 expression. TLR4 protein expression was evaluated in control BV-2 cells, BV-2 cells + HIF-1α Ab, BV-2 cells + hypoxia and BV-2 cells pretreated with HIF-1α Ab + hypoxia by western blot. The upper panel shows the specific band of TLR4 (95 kDa) and β-actin (43 kDa). The lower panel bar graph shows significant changes in the optical density in different groups mentioned above (given as fold change of control). Note that the increased TLR4 expression after hypoxia in BV-2 cells is significantly depressed with HIF-1α neutralization. *P <0.05 and **P <0.01. The values represent the mean ± SD in triplicate.

We have shown that hypoxia modulates TLR4 expression in microglia. However, the underlying mechanism of TLR4 expression in hypoxic BV-2 cells remains unclear. It has previously been reported that hypoxic stress upregulates the expression of TLR4 in macrophages via HIF-1, of which the main subunit, HIF-1α, binds to the TLR4 promoter region under hypoxic conditions [16]. To determine the mechanism of TLR4 increase in hypoxic microglia, we next investigated the role of HIF-1α in the regulation of TLR4 expression in hypoxic BV-2 cells. Earlier, we showed that HIF-1α mRNA expression level was not significantly affected but that of the protein expression was significantly increased in hypoxic BV-2 cells compared with the control (Figure 4). Hence, we inhibited HIF-1α protein function using HIF-1α antibody in BV-2 cells. Western blot analysis showed that TLR4 expression level, which was increased after hypoxic exposure for 8 h, was significantly reduced when hypoxic cells were pretreated with HIF-1α antibody and returned to control levels (Figure 9).

### NF-κB pathway was involved in TLR4 mediated microglial activation after hypoxic stress

**Figure 10:**
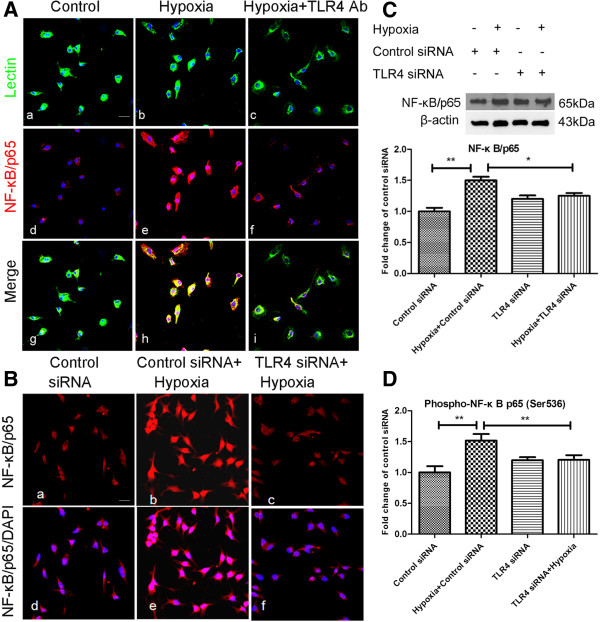
**Suppression of Toll-like receptor 4 (TLR4) with TLR4 antibody or siRNA inhibited Nuclear factor kappa-light-chain-enhancer of activated B cells (NF-κB) activation induced by hypoxic stress in primary microglia and BV-2 cells.** Immunofluorescence images showing NF-κB/p65 expression in primary microglia (**A**) and BV-2 cells (**B**). NF-κB/p65 is mainly localized in the cytoplasm of control microglia colocalized with lectin (Aa, Ad, Ag) and control siRNA-transfected BV-2 cells (Ba, Bd). The expression is intensely augmented both in the cytoplasm and nucleus after hypoxic treatment in both cell groups (Ae, Ah; Bd, Be). NF-κB/p65 immunoreactivity in the cytoplasm and nucleus after hypoxic exposure in primary microglia with TLR4 Ab (Af, Ai) is noticeably suppressed. A similar decrease is observed in TLR4 siRNA-transfected BV-2 cells subjected to hypoxic exposure (Bc, Bf). (**C**) Western blot analysis of NF-κB/p65 protein expression in BV-2 cells of different groups. The upper panel shows specific bands of NF-κB/p65 (65 kDa) and β-actin (43 kDa) and the lower panel bar graph shows significant changes in the optical density of different groups (given as fold change of control BV-2 cells). Note the NF-κB/p65 protein expression, which is increased after hypoxic exposure in control siRNA-transfected BV-2 cells, is significantly decreased after hypoxic exposure in TLR4-transfected groups. (**D**) ELISA analysis of phospho-NF-kB/p65 in different groups of BV-2 cells showing a similar trend as in **C**. *P <0.05 and **P <0.01. The values represent the mean ± SD in triplicate. Scale bars in **A** and **B** = 20 μm.

NF-κB activation has been reported to be involved in TLR4 signaling in microglia. NF-κB is an essential transcription factor for cytokines and iNOS expression in microglia after hypoxic treatment [22]. To gain further insight into the downstream effects of TLR4 expression in hypoxic microglia, we sought to determine whether the NF-κB pathway activation is responsible for the target gene expression induced by TLR4 after hypoxic treatment in microglia. This study confirmed that hypoxia induced the activation of the NF-κB pathway in microglial cells both in primary cultured (Figure 10A) and in BV-2 cells (Figure 10B).

In BV-2 cells, western blotting analysis indicated a significant increase in NF-κB in control siRNA-transfected BV-2 cells exposed to hypoxia when compared with the corresponding control. The increase in NF-κB expression was significantly prevented when BV-2 cells with TLR4 knockdown were exposed to hypoxia (Figure 10C). Additionally, the localization of the p65 subunit of NF-κB was assessed by immunofluorescence. Hypoxic exposure was found to increase the expression of the NF-κB subunit p65 in both the nucleus and cytoplasm, whereas NF-κB p65 was localized primarily in the cytoplasm in microglia during the resting state. Importantly, the immunofluorescence intensity of NF-κB p65 in both the nucleus and cytoplasm was reduced in the hypoxic TLR4 knockdown BV-2 cells compared with the hypoxic non-transfected and scramble siRNA-transfected BV-2 cells (Figure 10B). Similar results were observed in primary cultured microglia, in which the NF-κB subunit p65 expression in both the nucleus and cytoplasm was reduced in cells with blockade of TLR4 by antibody neutralization compared with the strong immunofluorescence intensity in the nucleus and cytoplasm induced by hypoxic exposure (Figure 10A). ELISA analysis of phospho-NF-kB p65 proteins showed that the phospho-NF-kB p65 protein expression was increased 1.5-fold following hypoxia, but the increase was inhibited in hypoxic BV-2 cells with TLR4 knockdown, confirming the results derived from western blot analysis and immunofluorescence (Figure 10D).

### TLR4 inhibition attenuated the expression of inflammatory mediators in neonatal rats following hypoxic injury

**Figure 11:**
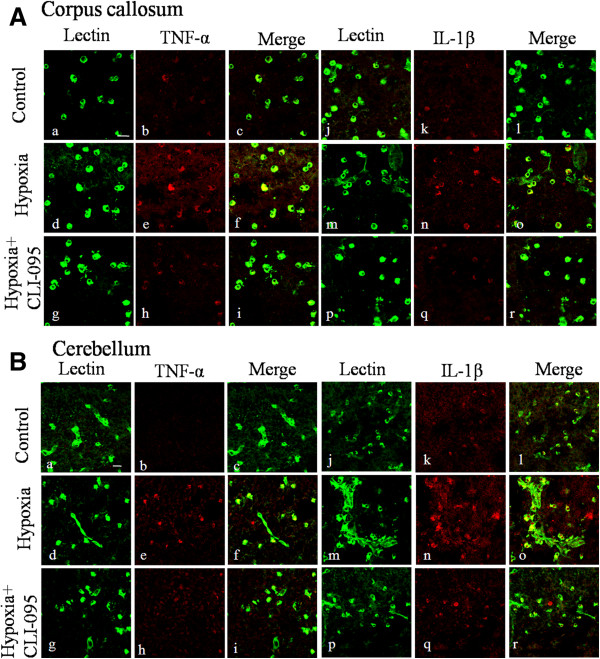
**Toll-like receptor 4 (TLR4) inhibition suppressed the increase in TNF-α and IL-1β immunoexpression on microglia in the corpus callosum and cerebellum in neonatal rats after hypoxic treatment.** Confocal images showing the expression of TNF-α and IL-1β in lectin-labeled (green) microglia in the corpus callosum (**A**) and cerebellum (**B**) of control, hypoxia and hypoxia + CLI-095 rats at 3 days after the hypoxic exposure. (**A**) Increase in TNF-α and IL-1β expression in microglia of the corpus callosum was evident in hypoxic rats. In hypoxia + CLI-095 rats, both increase in TNF-α and IL-1β was inhibited compared with the hypoxia rats. (**B**) TNF-α was undetected in the cerebellar microglia of normal rats (b) but its immunoexpression was intensely induced in hypoxic rats, while in hypoxia + CLI-095 rats the immunofluorescence intensity was attenuated. IL-1β immunofluorescence intensity was enhanced after hypoxia but the increase was inhibited in hypoxia + CLI-095 rats. Scale bars in **A** and **B** = 20 μm.

**Figure 12:**
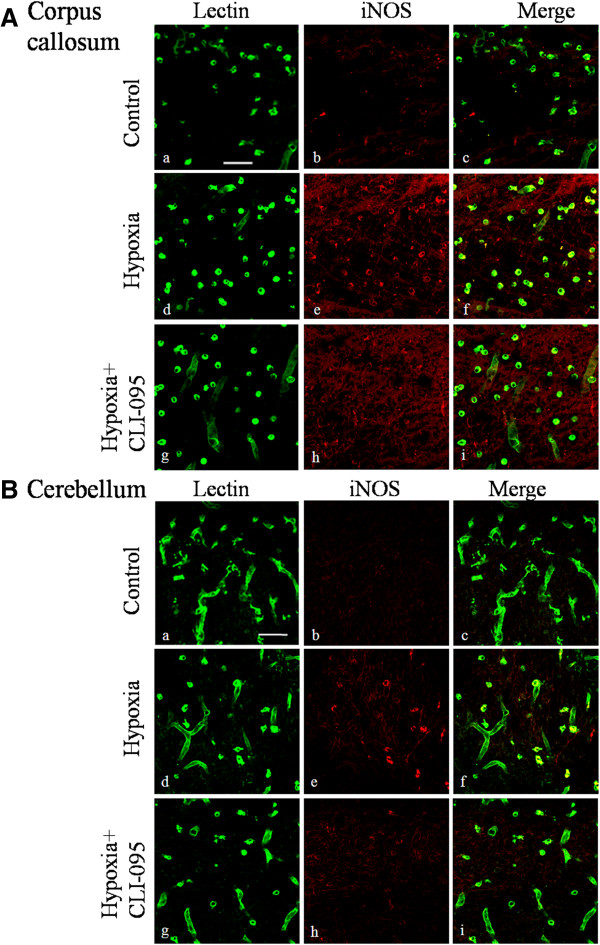
**Toll-like receptor 4 (TLR4) inhibition suppressed the increased inducible nitric oxide synthase (iNOS) expression on microglia in the corpus callosum and cerebellum in neonatal rats after hypoxic treatment.** Confocal images showing the expression of **i**NOS expression in lectin-labeled (green) microglia in the corpus callosum (**A**) and cerebellum (**B**) of control, hypoxia and hypoxia + CLI-095 rats at 3 h after the hypoxic exposure. (**A**) iNOS expression was barely detected on microglia of control rats; however, it was markedly enhanced in hypoxic rats. The expression was decreased on microglia of hypoxia + CLI-095 rats compared with the hypoxic rats. (**B**) A similar phenomenon was observed in microglia in the cerebellum. Scale bars in **A** and **B** = 50 μm.

To investigate the effect of TLR4 in the hypoxia-induced neuroinflammation *in vivo*, a specific TLR4 inhibitor CLI-095 was administrated into the neonatal rats before hypoxic exposure. Immunoexpression of TNF-α, IL-1β and iNOS on microglia both in corpus callosum and cerebellum was investigated by double immunofluorescence staining. Increase in TNF-α and IL-1β expression in microglia in the corpus callosum and cerebellum was observed at 3 days after the hypoxic exposure but the increase was noticeably suppressed in TLR4-inhibited rats (Figure 11). In comparison to TNF-α and IL-1β, iNOS immunoexpression in microglia appeared to be transient and was most intense at 3 h after hypoxia both in the corpus callosum and cerebellum. Based on this time point, an obvious attenuation in iNOS immunofluorescence intensity on microglia in CLI-095-injected rats was confirmed when compared with rats subjected to hypoxia only (Figure 12).

## Discussion

Microglial cells are the primary immune effector cells in the brain and play a pivotal role in the neuroinflammatory processes associated with a variety of neurological and pathological disorders [23]. We have reported that microglia are involved in hypoxic injuries in the developing brain, affecting both neurons and oligodendrocytes [1,6,24]. Hence, a fuller understanding of the underlying molecular mechanisms of microglial activation under such conditions would be desirable for the design of a novel therapeutic strategy for the management of hypoxic damage.

It is well documented that TLR4 in microglia is a key player of neuroinflammation in several neurodegenerative and CNS trauma diseases [12-14,25]. The data presented herein provide the first evidence of the role of microglial TLR4 in a neonatal rat model of hypoxic injury. It is unequivocal from the present results that TLR4 signaling participates in the microglial activation in the hypoxic developing brain. TLR4 immunoexpression was localized in microglia in the neonatal rat corpus callosum and cerebellum and was increased in hypoxia. *In vitro* studies further demonstrated that TLR4 expression in hypoxic microglia was dependent on HIF-1α and more importantly, that TLR4 mediates the production of inflammatory mediators through the NF-κB pathway. The effect of TLR4 as demonstrated *in vitro* was further confirmed *in vivo* by TLR4 inhibition with its specific inhibitor, namely, CLI-095. It is therefore suggested that this TLR4 pathway activation contributes to the neonatal brain damage resulting from hypoxic exposure.

Hypoxia results in changes in the signaling pathways and gene expression related to physiological as well as pathological responses. We have shown that TLR4 in microglia was overexpressed in the cerebrum and cerebellum of neonatal rats after hypoxic treatment. Very interestingly, many *in vivo* experimental and clinical studies have similarly reported that TLR4 expression is elevated in cells and tissues in hypoxia-related disease. The levels of TLR4 mRNA and protein have been found to be increased in murine hearts after myocardial ischemic injury, and in human hearts derived from patients with dilated cardiomyopathy and myocarditis [26,27]. In addition, TLR4 expression is significantly increased in Kupffer cells in rat liver grafts, and in tubular epithelial cells and infiltrating leucocytes within the kidney following ischemia [28,29]. Here, we have shown that TLR4 protein expression in primary microglia subjected to hypoxic exposure for 24 h was increased. In BV-2 cells, TLR4 protein overexpression was sustained with long-term hypoxic exposure from 4 to 12 h compared with the significant increase in mRNA expression from 2 to 6 h hypoxia exposure (data not shown). Consistent with our result, the increase in TLR4 protein expression in BV-2 microglial cells or primary microglial cultures is also reported with exposure to an extremely low concentration of oxygen (O_2_ tension <0.2%) for 8 or 24 h [17]. Hypoxic stress induced either by a low oxygen tension or CoCl_2_ can also enhance TLR4 expression in macrophages [16]. However, some studies report that relatively long-term hypoxia for 48 to 72 h diminishes TLR4 expression as a result of mitochondrial generation of ROS in human umbilical vein endothelial cells [30]. It would appear, therefore, from the above studies that differences in duration of hypoxic exposure and the type of cells investigated may account for the discrepancy in TLR4 expression over time.

It is widely demonstrated that exogenous ligands to TLR4 such as lipopolysaccharide (LPS) and endogenous ligands to TLR4, such as members of the heat shock protein family and proteoglycans, can lead to the induction of proinflammatory cytokines [20,31]. Intracerebral injection of LPS into the developing periventricular white matter of immature rodents results in the loss of oligodendrocytes, hypomyelination and formation of periventricular cysts through the action of TLR4 [32]. Hence, a hypothesis that TLR4 mediates the production of proinflammatory cytokines, which in turn, contributes to damage in the developing brain after hypoxia, was formed. To test this hypothesis, we adopted siRNA transfection and antibody neutralization in BV-2 cells and primary cultured microglia, separately, in the present study. We show here that inhibition of TLR4 expression in microglia effectively suppressed the overexpression of inflammatory factors elicited by hypoxic stress. This suggests that increased microglial TLR4 activation correlates with the production of inflammatory factors after hypoxic stress, even in the absence of exogenous TLR4 ligands such as LPS. A similar function of TLR4 has also been reported in microglia stimulated with other factors. For example, we have reported previously that TLR4 signaling mediated the inflammation in microglia that was stimulated with saturated fatty acid [33]. It has also been reported that TLR4 is upregulated in ethanol-induced microglial activation and response, suggesting that the activation of microglial TLR4 signaling could trigger the release of inflammatory mediators, which in turn could induce white matter abnormalities and neuronal dysfunctions [13]. Accumulating evidence has shown a general participation of TLR4 in the neuroinflammatory process and brain damage of different pathological factors, suggesting that TLR4 exacerbates brain damage in neuroinflammatory diseases. In contrast, studies with TLR4 mutation mice indicate that microglia are activated via TLR4 signaling to reduce β-amyloid deposits and preserve cognitive functions from β-amyloid-mediated neurotoxicity [12]. In this case, activation of microglia through TLR4 appears to be neuroprotective in Alzheimer’s disease. Therefore, whether TLR4 is neuroprotective or neurotoxic in neuroinflammatory disorders may vary with different pathological conditions and the different stages of these diseases. TLR4 activation appears to be tightly controlled, in order to regulate the switch between its different roles in immune surveillance or neuroinflammatory propagation. The present results tend to support the theory that TLR4 promotes the production of major inflammatory mediators such as TNF-α, IL-1β, iNOS, ROS and NO, which have been implicated in oligodendrocyte and neuronal death, thus, suggesting its detrimental role in hypoxic neonatal brain injury.

The identification of factors responsible for the induction of TLR4 expression in microglia under hypoxia has remained elusive. HIF-1 has been reported to mediate the TLR4 expression in macrophages under hypoxic conditions [16]. This has prompted us to determine the role of HIF-1 in TLR4 expression in hypoxic microglia. HIF-1 is a major transcription factor activated during hypoxia in pathological conditions and it plays critical roles in inducing hypoxia-related gene expression and cellular responses. HIF-1 is a heterodimeric protein that consists of HIF-1β and HIF-1α, among which HIF-1β is constitutively expressed while HIF-1α expression is tightly regulated by oxygen [34,35]. Therefore, the overall activity of HIF-1 is dictated by the intracellular HIF-1α level. In this study, a hypoxia model was first achieved in BV-2 cells by placing the cells in a chamber filled with a gas mixture of 3% O_2_, 5% CO_2_ and 92% N_2_ for 0.5 h or more followed by assessing the HIF-1α protein expression. Remarkably, there was no significant difference in HIF-1α mRNA expression level between the hypoxic and control cells. On the other hand, HIF-1α protein expression was significantly increased in hypoxia. It has been reported that HIF-1α is degraded rapidly by the hydroxylation of prolyl hydroxylase, and the enzymatic activity of prolyl hydroxylase is oxygen-dependent. Under hypoxic conditions, the enzymatic activity of prolyl hydroxylase is significantly reduced, resulting in the increase in HIF-1α [36,37]. Our findings of increased HIF-1α protein expression in hypoxic BV-2 cells is in accordance with the mechanism of HIF-1α increase in hypoxic conditions. A major finding in this study was that TLR4 expression was regulated by HIF-1α in hypoxic microglia. This is evidenced by the fact that TLR4 expression decreased on neutralization of HIF-1α with its antibody. Indeed, several recent studies support the notion of the link between HIF-1α and TLR4. Studies in macrophages found that TLR4 expression is upregulated via HIF-1α in response to hypoxic stress. Interestingly, some studies also found that LPS, a ligand of TLR4 can induce accumulation and DNA-binding activity of HIF-1α protein in murine microglial cells [38], macrophage-differentiated cells [39] and macrophages [40]. In other words, LPS can raise the level of HIF-1α in a TLR4-dependent fashion, suggesting that TLR4 may act conversely to regulate HIF-1α expression. Positive correlation between TLR4 and HIF-1α has also been found in pancreatic ductal adenocarcinoma cells indicating that there may exist a crosstalk between the TLR4 signal pathway and the HIF-1 signal pathway, which may act synergistically to promote the progression of pancreatic ductal adenocarcinoma [41]. We have demonstrated the role of HIF-1α in TLR4 expression in hypoxic microglia; but it remains to be elucidated whether the two act synergistically.

NF-κB is a transcription factor known to regulate genes of a spectrum of processes such as inflammation, stress responses, innate, and acquired immunity [42,43]. Under normal physiological conditions NF-κB is localized in the cytoplasm in an inactive state bound to Inhibitor of nuclear factor-κB (IκB). Under pathological conditions IκB is phosphorylated and degraded, resulting in active NF-κB [44]. Activated NF-κB is in turn phosphorylated and subsequently translocated to the nucleus where it regulates target gene expression. It has been reported that hypoxia induces rapid degradation of phosphorylated IκB and an increase in phosphorylated NF-κB in hypoxic microglia of neonatal rats [45]. Activation of the NF-κB pathway through the TLR4 pathway has been reported not only in LPS-treated but also in non-TLR4 ligands, such as fatty acid-treated microglia [17,33]. The present results showed nuclear translocation of NF-κB in hypoxic BV-2 microglial cells, and more importantly, the translocation was prevented by TLR4 knockdown. Moreover, the increase in phosphorylated NF-κB expression, which was increased after hypoxic exposure, was inhibited after TLR4 knockdown. Results with primary cultured microglia are in accordance with the observations made in BV-2 cells. The present results suggest that TLR4 mediates the activation/nuclear translocation of NF-κB in microglia after hypoxia stress. In contrast, it has been reported that hypoxia suppressed LPS-induced NF-kB activation in microglia [17]. It is suggested that the mechanism involved in cells treated with hypoxia alone may be different from LPS stimulation along with hypoxic exposure. Although our results have shown that NF-κB signaling represents one of the downstream pathways of TLR4-induced production of inflammatory factors, the possibility of involvement of other pathways such as mitogen-activated protein kinase (MAPK) [46] and Notch pathways in hypoxia-induced inflammation (unpublished data for Notch pathway) should also be considered.

CLI-095, a specific inhibitor of TLR4, also called TAK 242, has been reported to specifically and effectively inhibit TLR4 signaling in different animal models [47-50]. We have demonstrated the effect of TLR4 inhibition on microglial activation *in vivo* and this has further confirmed the role of TLR4 in neonatal brain damage following hypoxia. Very interestingly, the protective effect observed with microglia in both the cerebrum and cerebellum indicates that TLR4 may mediate in damage in both oligodendrocytes and Purkinje neurons. Indeed, obvious reduction in apoptosis of oligodendrocytes and Purkinje neurons was found after TLR4 blockade in hypoxic rats (data not shown), although direct evidence for the contribution of the inhibition of the activation of microglia awaits further investigation. Notwithstanding, it can be confidently concluded from the present results that TLR4 inhibition can effectively inhibit release of inflammatory mediators by microglia in hypoxic neonatal rats.

## Conclusions

The present findings have highlighted that TLR4 signaling regulates the expression of hypoxia-induced inflammatory mediators via the NF-κB pathway and is linked to the inflammatory process in neonatal hypoxic brain injury. Thus, inhibiting the activation of microglia via TLR4 in neonatal hypoxic injury may be neuroprotective, for which the TLR4 signaling pathway represents a potential therapeutic target.

## Abbreviations

AD: Alzheimer’s disease; AMC: Amoeboid microglial cell; ANOVA: Analysis of variance; BBB: Blood–brain-barrier; CNS: Central nervous system; DAPI: 4^′^,6-diamidino-2-phenylindole; DMEM: Dulbeco’s Modified Eagle’s Medium; DMSO: Dimethyl sulfoxide; ELISA: Enzyme-linked immunosorbent assay; FBS: Fetal bovine serum; HIF-1α: Hypoxia-inducible factor-1 alpha; IκB: Inhibitor of nuclear factor-κB; IL-1β: Interleukin-1 beta; iNOS: Inducible nitric oxide synthase; LPS: Lipopolysaccharide; MAPK: Mitogen-activated protein kinase; NF-κB: Nuclear factor kappa-light-chain-enhancer of activated B cells; NO: Nitric oxide; PBS: Phosphate-buffered saline; PD: Parkinson’s disease; PWM: Periventricular white matter; PWMD: Periventricular white matter damage; ROS: Reactive oxygen species; RT-PCR: Reverse transcription polymerase chain reaction; siRNA: Small interfering RNA; TLR4: Toll-like receptor 4; TNF-α: Tumor necrosis factor-alpha.

## Competing interests

The authors declare that they have no competing interests.

## Authors’ contributions

EAL designed the project. CK and AH contributed to the analysis of data and finalization of the manuscript. LY conducted all experiments and prepared the first draft of the manuscript. EMK, JL and STD participated in discussion and analysis of data as well as editing the manuscript. All authors have read and approved the final version of the manuscript.
